# A new fluorescent chemosensor for fluoride anion based on a pyrrole–isoxazole derivative

**DOI:** 10.3762/bjoc.7.8

**Published:** 2011-01-12

**Authors:** Zhipei Yang, Kai Zhang, Fangbin Gong, Shayu Li, Jun Chen, Jin Shi Ma, Lyubov N Sobenina, Albina I Mikhaleva, Guoqiang Yang, Boris A Trofimov

**Affiliations:** 1 Beijing National Laboratory for Molecular Sciences, CAS Key Laboratory of Photochemistry, Institute of Chemistry, Chinese Academy of Sciences, Beijing, 100190, China; 2 A. E. Favorsky Irkutsk Institute of Chemistry, Siberian Branch of the Russian Academy of Sciences, 1 Favorsky Str., Irkutsk, 664033, Russian Federation

**Keywords:** deprotonation, fluorescent chemosensor, fluoride anion recognition

## Abstract

Molecules containing polarized NH fragments that behave as anion-binding motifs are widely used as receptors for recognition and sensing purposes in aprotic solvents. We present here a new example of a receptor, 3-amino-5-(4,5,6,7-tetrahydro-1*H*-indol-2-yl)isoxazole-4-carboxamide (receptor **1**), which contains pyrrole, amide and amino subunits. This receptor shows both changes in its UV–vis absorption and fluorescence emission spectra upon the addition of F^−^, resulting in highly selectivity for fluoride detection over other anions, such as Cl^−^, Br^−^, I^−^, HSO_4_^−^, H_2_PO_4_^−^ and AcO^−^ in CH_3_CN. ^1^H NMR titration, time-dependent density functional theory (TDDFT) calculations and other experiments confirm that the sensing process is brought about by deprotonation of the pyrrole-NH in receptor **1**.

## Introduction

The development of anion receptors has become a field of substantial interest and activity [1–3]. Among the various artificial receptors reported in recent years, those employing polarized NH groups as anion-binding motifs have attracted considerable attention. Typical examples are charge neutral receptors containing pyrrole, amide, indolocarbazole, guanidium, imidazolium and urea/thiourea moieties. Usually, the anions are recognized via H-bonding, which is not easy to differentiate from deprotonation of protons on the receptor-NH [4–5]. Some urea/thiourea-containing receptors could particularly recognize Y-shaped oxoanions by H-bonding and more basic anions such as fluoride by deprotonation. Fluoride is primarily used for prevention of dental caries [6], enamel demineralization while wearing orthodontic appliances, and in treatment of osteoporosis [7–8]. However, excessive fluoride ingestion can cause skeletal and dental injuries, nephrotoxic changes in both humans and animals, and lead to urolithiasis. Hence, it is highly advantageous to develop high-effective sensors that can detect fluoride anion in food and animal feed.

**Figure 1:**
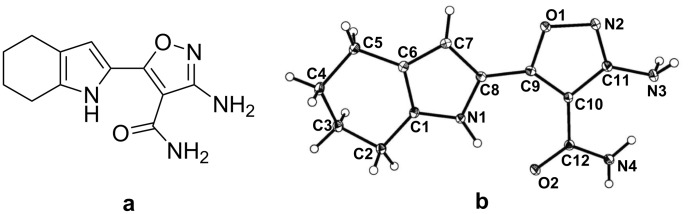
(a) Receptor **1**. (b) ORTEP drawing of receptor **1.** Thermal ellipsoids are drawn at the 30% probability level.

In this work, we report a new fluoride receptor **1** (Figure 1), 3-amino-5-(4,5,6,7-tetrahydro-1*H*-indol-2-yl)isoxazole-4-carboxamide [9], which contains receptive groups toward anions and no urea/thiourea moieties which avoids the problem of multi-anion sensitivity. The common anion recognition moieties, i.e., a pyrrole NH and an amide group, present in the structure behave as proton donors. Isoxazoles and their derivatives are important intermediates in preparation of many natural products and related compounds, and are used as antimicrobial antifungal and herbicide agents [10–11]. Research on the mechanism of anion recognition is helpful for understanding the biological activities of pyrrole–isoxazole derivatives. From UV–vis and fluorescence titration experiments it was found that the receptor **1** could recognize fluoride anion (F^−^) with high selectivity and sensitivity over other anions (Cl^−^, Br^−^, I^−^, HSO_4_^−^, H_2_PO_4_^−^ and AcO^−^). Both ^1^H NMR titration experiments and time-dependent density functional theory (TDDFT) calculations demonstrated that the mechanism is deprotonation of the pyrrole-NH.

## Results and Discussion

### Anion-sensing in CH_3_CN solution

**Figure 2:**
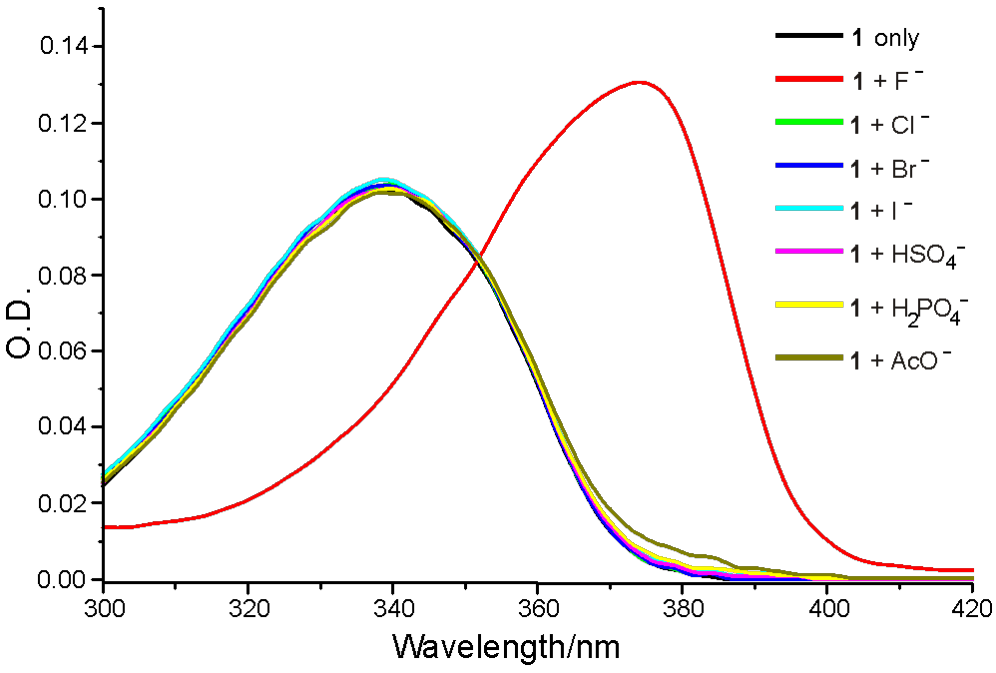
The absorption spectra of receptor **1** (5 μM) in the absence or presence of a 50 equiv of F^−^, Cl^−^, Br^−^, I^−^, HSO_4_^−^, H_2_PO_4_^−^ and AcO^−^ anions in CH_3_CN.

**Figure 3:**
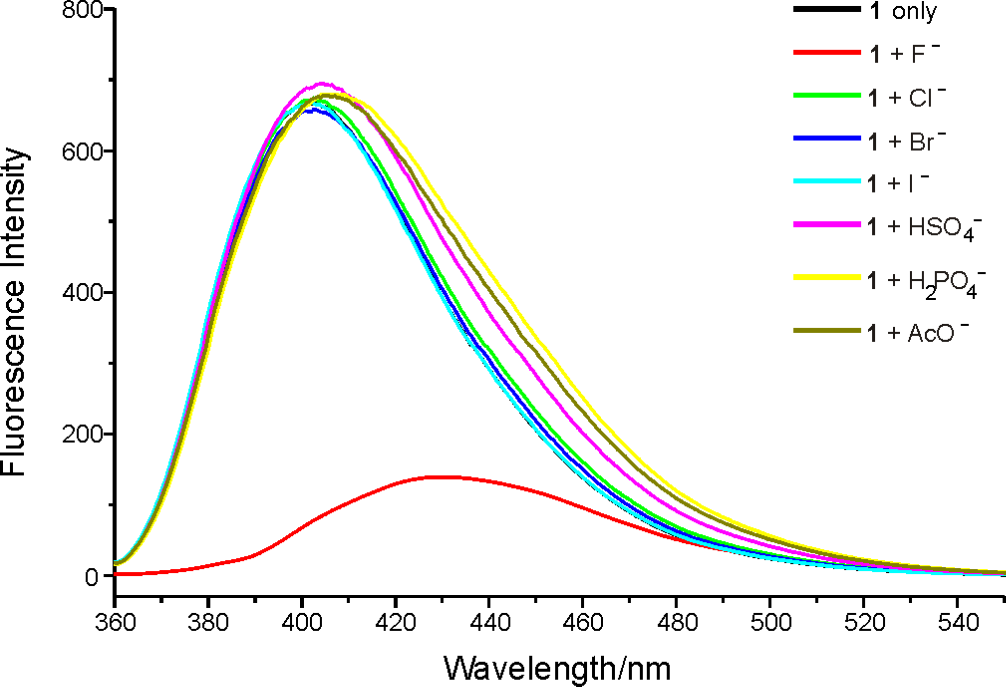
The fluorescence spectra of receptor **1** (5 μM) in the absence or presence of a 50 equiv of F^−^, Cl^−^, Br^−^, I^−^, HSO_4_^−^, H_2_PO_4_^−^ and AcO^−^ anions in CH_3_CN (excited at 340 nm).

The selectivity of receptor **1** for F^−^ anion over other anions was studied. Variations in the UV–vis absorption spectra and fluorescence spectra of **1** in acetonitrile (5 μM) in the presence of anions such as F^−^, Cl^−^, Br^−^, I^−^, HSO_4_^−^, H_2_PO_4_^−^ and AcO^−^ (50 equiv as their tetrabutylammonium salts) are shown in Figure 2 and Figure 3, respectively. It was found that, whereas receptor **1** exhibited only an absorption peak at 340 nm in CH_3_CN, a new and red-shifted absorption appeared at 375 nm when the F^−^ ion was added. Other anions such as Cl^−^, Br^−^, I^−^, HSO_4_^−^, H_2_PO_4_^−^ and AcO^−^ did not produce any change under the same conditions.

The receptor **1** displayed an emission maximum at 400 nm with a fluorescence quantum yield of 0.067 (determined by comparison with 1,4-bis(5-phenyl-2-oxazolyl)benzene as the reference compound, similarly in all other cases) [12] when excited at 340 nm. The changes in fluorescence intensity of **1** upon the addition of particular anions are shown in Figure 3. This clearly shows that the fluorescence intensity was remarkably quenched and the emission peak red shifted from 400 nm to 432 nm upon the addition of F^−^, however no significant quenching was observed on the addition of other anions.

**Figure 4:**
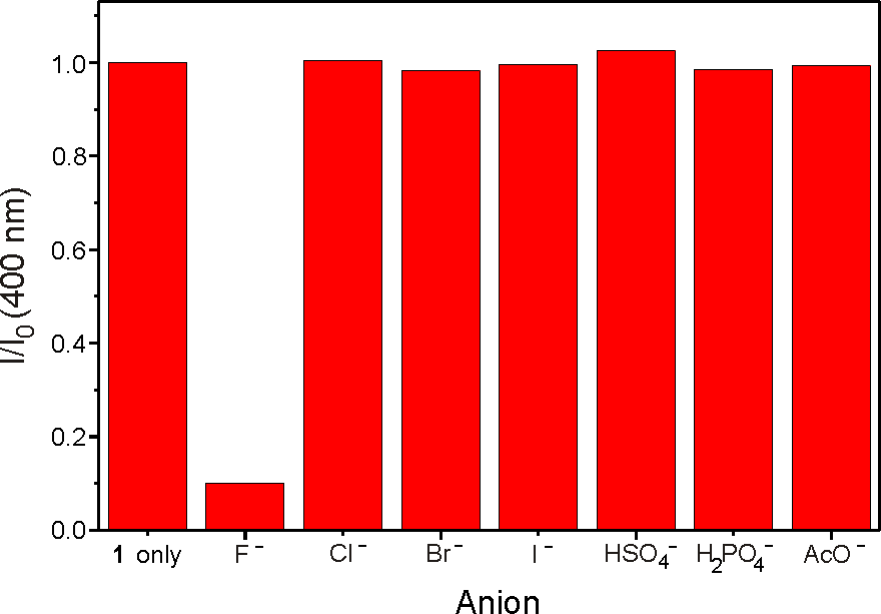
Comparison of fluorescence emission of **1** (5 μM) in CH_3_CN after the addition of 50 equiv of tetrabutylammonium salts.

For some receptors, the presence of H_2_PO_4_^−^ and AcO^−^ interferes with the detection of F^−^ [13]. However, in the case of receptor **1**, addition of the same equiv amount of H_2_PO_4_^−^ or AcO^−^, as well as other anions such as Cl^−^, Br^−^, I^−^ and HSO_4_^−^, did not lead to quenching of the fluorescent emission (Figure 4). The results indicate that the receptor **1** is a good sensor for recognizing F^−^ over other anions.

**Figure 5:**
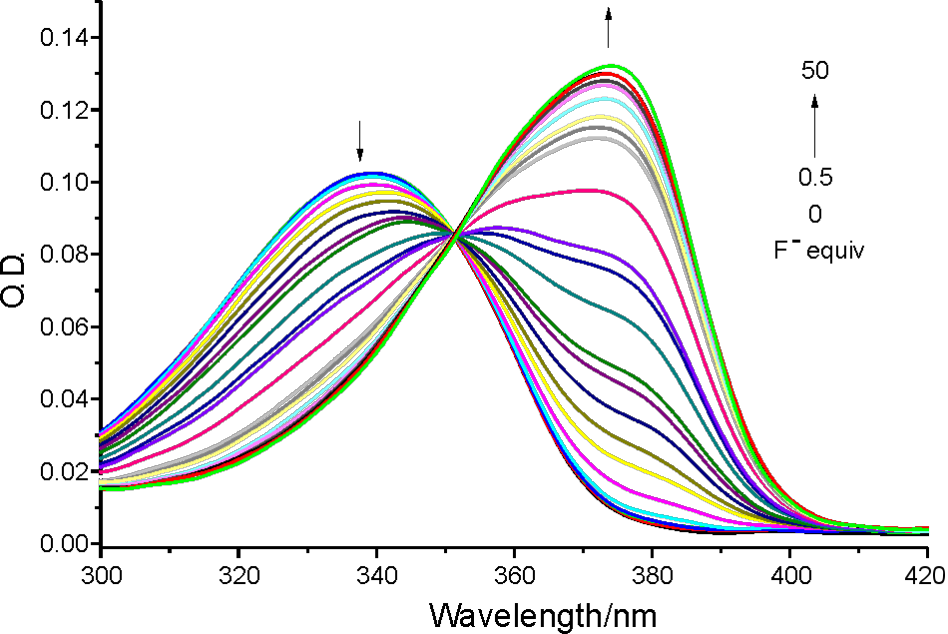
UV–vis absorption changes of **1** (5 μM) upon the addition of TBAF in CH_3_CN.

**Figure 6:**
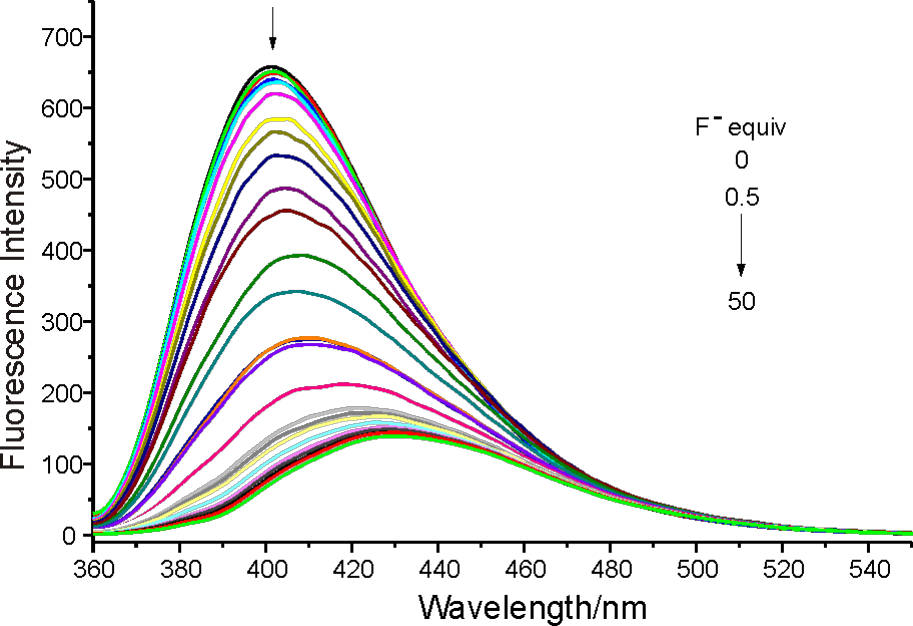
Fluorescence emission changes of **1** (5 μM) upon the addition of TBAF in CH_3_CN (excited at 340 nm).

The F^−^ titration experiments were carried out in CH_3_CN for further investigation (Figure 5, Figure 6). With increasing F^−^ concentration, the absorbance of **1** at 340 nm decreased significantly and a new adsorption peak appeared at 375 nm with a sharp isosbestic point formation at 352 nm, which indicated that only two species are present in the equilibrium throughout the titration process [14]. The absorption peak at 375 nm in CH_3_CN was partially returned to 340 nm when a protic solvent such as methanol or water was introduced, which suggested that the interaction of **1** and F^−^ is due to hydrogen bonding [15] or deprotonation [16]. Because of the greater contribution of the electron density to the conjugated system in the deprotonated **1** it could be concluded that the interaction is deprotonation rather than the formation of hydrogen bond.

**Figure 7:**
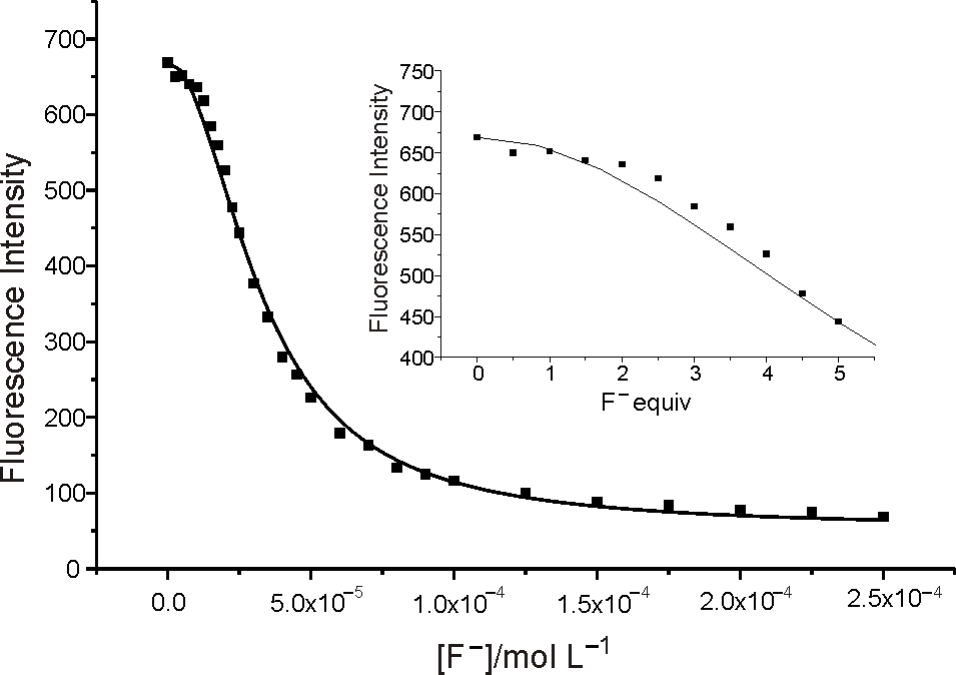
The fit of the experimental data of fluorescence emission of **1** (5 μM) upon the addition of F^−^ at 400 nm to a 1:2 binding profile (excited at 340 nm). Inset: the partial enlarged curve when less than 5 equiv of F^−^ was added.

Figure 6 shows the changes of fluorescence emission of **1** upon addition of F^−^ in CH_3_CN where the emission maximum was red-shifted to 432 nm. With increasing F^−^ concentration, the emission intensity was quenched by about 90% when 50 equiv of F^−^ was added. The quantum yield of fluorescence was reduced to 0.031 in this case. The stoichiometry of the equilibrium was found to be 1:2 by the existence of the inflexion in the titration profile (insert) at 400 nm (Figure 7). Usually, the deprotonation of an NH moiety caused by F^−^ includes two steps. The first step is the formation of a 1:1 stoichiometry host–guest complex through hydrogen bonding; the second step is the deprotonation of the host with the formation of **1^−^** anion and HF_2_^−^ self-complex, as illustrated in equilibria 1 and 2:

[1]
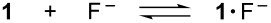


[2]
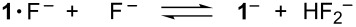


The fluorescence intensity of **1** was not significant changed when less than 2 equiv of F^−^ was added, which suggested the formation of hydrogen-bond complex of **1**·F^−^. However, the fluorescence intensity decreased drastically on further increasing the F^−^ concentration, which indicated that such a hydrogen-bond complex interacted further with F^−^ in the formation of **1**^−^ anion and HF_2_^−^. The stoichiometry of the total equilibrium could be determined by fitting the experiment data as being 1:2 between **1** and F^−^; the same results were also obtained from the profile spectra. The stability constant of the two steps were obtained at the same time and were log K_1_ = 2.58 ± 0.15 and log K_2_ = 6.38 ± 0.15, respectively [17–18] (Figure 7).

### Studies on reaction with hydroxide (OH^−^)

**Figure 8:**
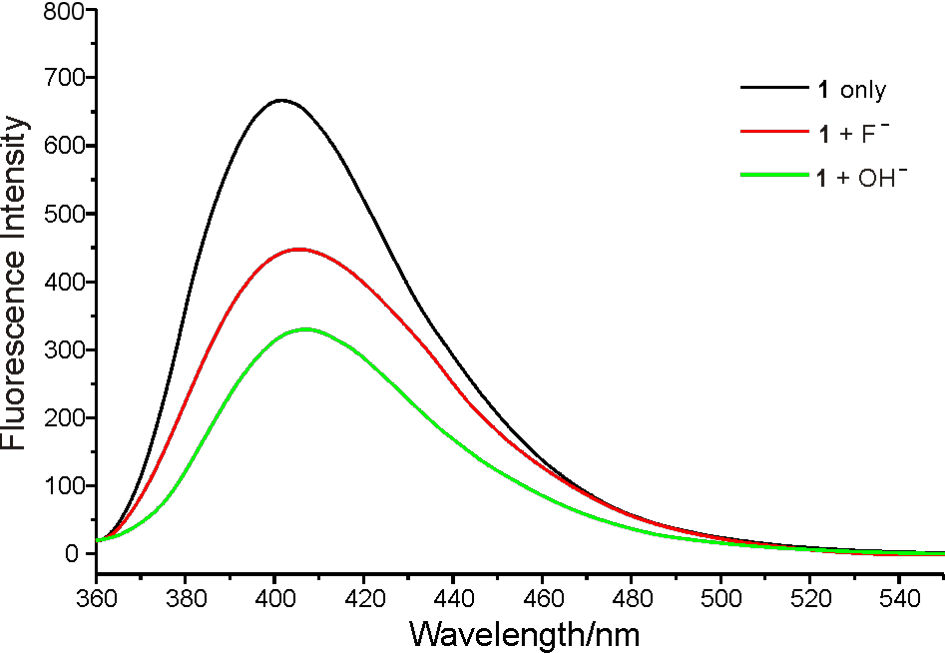
Fluorescence emission changes of **1** (5 μM) upon the addition of F^−^ and OH^−^ (5 equiv) in CH_3_CN (excited at 340 nm).

Tetrabutylammonium hydroxide was added to the solution of **1** in CH_3_CN to investigate the above process. Changes in fluorescence emission of **1** upon addition of F^−^ and OH^−^ in CH_3_CN were almost the same, except for the degree of quenching, as shown in Figure 8. Upon the addition of 5 equiv of OH^−^ the fluorescence emission of receptor **1** displayed λ_max_ at 408 nm and the intensity was quenched by about 51%. On the other hand, upon addition of F^−^ the emission displayed λ_max_ at 405 nm but the intensity was quenched by only about 33%, i.e., less than that caused by OH^−^. Considering the stronger basicity of OH^−^, it preferred to react with receptor **1** by deprotonation rather than by H-bonding [19]. The similar phenomena upon the addition of F^−^ and OH^−^ suggested that receptor **1** recognizes F^−^ in the same way as OH^−^.

### ^1^H NMR titration

**Figure 9:**
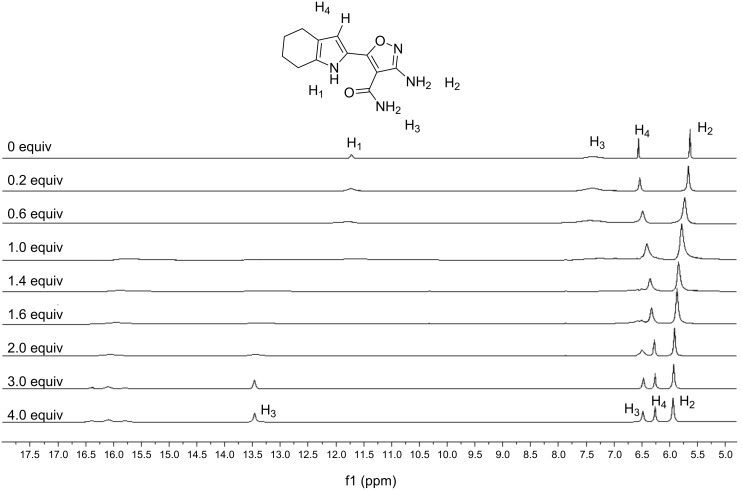
Partial ^1^H NMR (400 MHz) spectra of receptor **1** in the presence of 0, 0.2, 0.6, 1.0, 1.4, 1.6, 2.0, 3.0 and 4.0 equiv of TBAF in DMSO-*d*_6_.

^1^H NMR titration was also carried out to confirm the deprotonation of receptor **1** by F^−^. Figure 9 shows the series of ^1^H NMR spectra of **1** upon the addition of increasing amounts of TBAF in DMSO-*d*_6_. As discussed above, HF_2_^−^ anion was formed when the receptor was deprotonated by F^−^ via a two-step process. We found from the ^1^H NMR titration experiment that a new signal at 16.1 ppm (*J*_HF_ = 120 Hz), which was attributed to the HF_2_^−^ anion, appeared after 0.6 equiv of F^−^ was added [20]. However, the HF_2_^−^ anion could come from the deprotonation of either the NH group or of the solvent. However, the pyrrole CH proton signal (H_4_) was upfield shifted with increasing F^−^ concentration, reflecting the increase in electron density in the pyrrole ring [21]. This supports the hypothesis that the F^−^ preferably interacts with the receptor-NH rather than solvent molecules. Because of the higher charge density and smaller size, fluoride as strong base can deprotonate the receptor **1** to afford the heterocyclic conjugated anion [22–23], the emission of which is significantly lower than that of its charge neutral species when excited at 340 nm.

The deprotonation site could be determined by the ^1^H NMR titration spectra (Figure 9) upon the addition of a sufficient amount of fluoride. There are three types of NH proton signals in the free receptor **1**, which are designated as H_1_, H_2_ and H_3_, respectively. It was found that the pyrrole NH (H_1_) in the downfield part of the spectrum gradually broadened, weakened and finally disappeared with increasing F^−^ concentration. Meanwhile, the signal for amide NH (H_3_) also weakened and disappeared but gradually reappeared as two new signals at 6.5 ppm and 13.5 ppm after addition of 2.0 equiv of F^−^. However, the signal for the amino NH (H_2_) was only slightly downfield-shifted (0.31 ppm) with no broadening or weakening during the same process. Obviously, the disappearance of the pyrrole NH (H_1_) proton indicated clearly that the bifluoride signal at 16.1 ppm arose from the deprotonation of this NH moiety.

### Time-dependent density functional theory (TDDFT) calculation

**Figure 10:**
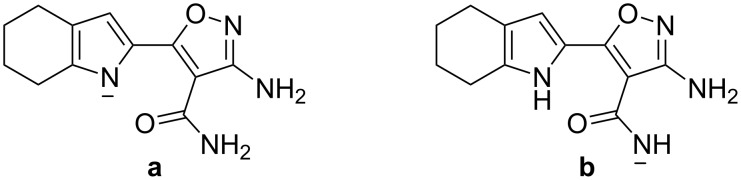
Anionic form **a** and **b** of receptor **1**.

To further investigate the chemical transformation of receptor **1** from neutral to its anionic form, the lowest energy electronic excited states of receptor **1** and its potential anionic forms were calculated at the B3LYP/6-31G(d) level using the TDDFT approach on their previously optimized ground-state molecular geometries in CH_3_CN [24–28]. Wavelengths and the oscillator strengths are listed in Table 1. The absorption λ_max_ of anionic forms **a** and **b** (Figure 10) were calculated since the pyrrole NH and amide NH are typical proton donors which are both easily deprotonated by strong base [29]. The calculated absorption wavelength of free receptor **1** was 338.5 nm, only 1.5 nm lower than the experimental value (340 nm) which suggests that TDDFT is suitable for calculating the absorption wavelengths of receptor **1** and its anionic forms. Thus the absorption wavelengths of its two anionic forms, **a** and **b**, were calculated to be 363.6 nm and 323.6 nm, respectively. The calculated absorption wavelength of anionic form **a** is much closer to the experimental result than that of anionic form **b**. Thus the NH fragment involved in the deprotonation process is the pyrrole NH rather than the amide NH. This conclusion is also in agreement with the results of the ^1^H NMR titration experiment.

**Table 1:** Calculated wavelengths (λ_max_), oscillator strengths of absorption spectra of receptor **1** and its anionic forms using TDDFT at level of B3LYP/6-31G(d) in CH_3_CN.

	λ_max_ (nm)	Oscillator strengths

receptor **1**	338.5	0.670
anionic form **a**	363.6	0.549
anionic form **b**	323.6	0.730

## Conclusion

In conclusion, we report a new fluorescent chemosensor, pyrrole–isoxazole derivative, for fluoride recognition in CH_3_CN. The UV–vis and fluorescence titration experiments revealed that the receptor-NH could be easily deprotonated by fluoride via a two-step process. This is confirmed by the appearance of the HF_2_^−^ anion in the ^1^H NMR titration experiment, and the pyrrole-NH is considered to be involved in the deprotonation process. The result of time-dependent density functional theory calculation also indicates that the mechanism of anion recognition is via the deprotonation of the pyrrole-NH.

## Experimental

**Materials**. Tetrabutylammonium salts were commercially available and were used without further treatment. Organic solvents were dried and distilled by appropriate methods before use. A detailed description of the synthesis of receptor **1** has been reported [5]. Crystals of receptor **1** suitable for analysis by single crystal X-ray diffraction were obtained by recrystallization from CH_3_CN.

**Methods**. UV–vis absorption spectra were recorded on a Hitachi U-3010 spectrophotometer. Fluorescence spectra were recorded on a Hitachi F-4500 fluorescence spectrophotometer. Fluorescence quantum yields (*Ф*_F_) were determined by the comparative method using 1,4-bis(5-phenyl-2-oxazolyl)benzene (*Ф*_F_ = 0.97) as reference standard [12]. ^1^H NMR spectra were recorded on a Bruker dmx 400 MHz NMR spectrometer at room temperature with DMSO-*d*_6_ as solvent and tetramethylsilane (TMS) as internal standard.

**Theoretical method.** Density functional theory (DFT) calculations were carried out by means of the Gaussian suite of programs to optimize the structure parameters of receptor **1** and its anionic forms. Becke’s three-parameter exchange functional combined with the LYP correlation functional (B3LYP) was employed because it has been shown that the B3LYP functional yields similar geometries for medium-sized molecules as MP2 calculations with the same basis sets. The standard 6-31G(d) basis set was used to obtain optimized geometries on isolated entities. The UV-vis absorption wavelengths (λ_max_), oscillator strengths of receptor **1** and its anionic forms were computed by the time-dependent DFT (TDDFT) approach at level of B3LYP/6-31G(d) in CH_3_CN.

**X-ray crystallography**: Accurate unit cell parameters were determined by a least-squares fit of 2*θ* values, measured for 200 strong reflections, and intensity data sets were measured on Rigaku Raxis Rapid IP diffractometer with Mo-*K*_α_ radiation (*λ* = 0.71073 Å) at room temperature. The intensities were corrected for Lorentz and polarization effects, but no corrections for extinction were made. All structures were solved by direct methods. The non-hydrogen atoms were located in successive difference Fourier synthesis. The final refinement was performed by full-matrix least-squares methods with anisotropic thermal parameters for non-hydrogen atoms on *F*^2^. The hydrogen atoms were added theoretically as riding on the concerned atoms.

CCDC reference number 755750.

## Supporting Information

File 1 Crystal data and structure refinement information for receptor **1**.
