# Supplementary material for: A new fluorescent chemosensor for fluoride anion based on a pyrrole–isoxazole derivative
Source: Beilstein J Org Chem. 2011 Jan 12;7:46–52. doi: 10.3762/bjoc.7.8 (PMC3028564; doi:10.3762/bjoc.7.8)
Supplement: File 1 — Crystal data and structure refinement information for receptor 1. [file Beilstein_J_Org_Chem-07-46-s001.pdf]

Supporting Information

for

A new fluorescent chemosensor for fluoride anion based on a pyrrole–isoxazole derivative

Zhipei Yang[1], Kai Zhang[1], Fangbin Gong[1], Shayu Li[1], Jun Chen[1], Jin Shi Ma[1], Lyubov N. Sobenina[2], Albina I. Mikhaleva[2], Guoqiang Yang*[,1] and Boris A. Trofimov*[,2]

Address: [1]Beijing National Laboratory for Molecular Sciences, CAS Key Laboratory of Photochemistry, Institute of Chemistry, Chinese Academy of Sciences, Beijing, 100190, China and [2]A. E. Favorsky Irkutsk Institute of Chemistry, Siberian Branch of the Russian Academy of Sciences, 1 Favorsky Str., Irkutsk, 664033, Russian Federation

Email: Guoqiang Yang - gqyang@iccas.ac.cn; Boris Trofimov - boris_trofimov@irioch.irk.ru

Crystal Data and Structure Refinement Information for Receptor 1

Table S1: Crystal and structure refinement data for receptor **1**

Receptor	**1**
empirical formula	$C_{14} H_{14}N_4O_2$
F_w	246.27
crystallizing solvent	CH_3CN
unit cell	Monoclinic
space group	P2(1)/c
T(K)	113(2)
dimensions	
a [Å]	13.341(3)
b [Å]	6.5946(13)
c [Å]	12.590(3)
angles	
α [°]	90
β [°]	93.03(3)
γ [°]	90
V [$Å^3$]	1106.0(4)
Z	4
F(000)	520
ρ [g cm^{-3}]	1.479
μ(mm^{-1})	0.105
Reflections collected	8100
Unique reflections	2634
R_{int}	0.0415
Final R indices [I>2σI]	R_1^a = 0.0447, wR_2^b = 0.1149
R indices (all data)	R_1 = 0.0572, wR_2 = 0.1327
GOF	1.130

[a] $R_1=\Sigma||F_0|-|Fc||/\Sigma|F_0|$, [b] $wR_2=[\Sigma w(F_0^2-Fc^2)^2/ \Sigma w(F_0^2)^2]^{1/2}$.
